# 4464 Effect of Surface Topography on *In Vitro* and Mechanical Performance of 3D Printed Titanium

**DOI:** 10.1017/cts.2020.386

**Published:** 2020-07-29

**Authors:** Bijan Abar, Cambre Kelly, Anh Pham, Nicholas Allen, Helena Barber, Alexander Kelly, Ken Gall, Samuel Adams

**Affiliations:** 1 Duke University

## Abstract

OBJECTIVES/GOALS: The goal of the study is to understand how changing the surface roughness of 3D printed Titanium either by processing printed samples or artificially printing rough topography impacts the mechanical and biological properties of the Titanium. METHODS/STUDY POPULATION: Titanium dog bones and discs were printed via laser powder bed fusion. groups were defined as 1. polished, 2.blasted, 4.as built, 4.sprouts and 5.rough sprouts. Roughness was measured with line measurement using a confocal microscope. Tensile testing of dog bones produced stress strain curves. MC3T3 preosteoblast were seeded on discs. Samples were analyzed at 0, 2, and 4 weeks. A cell viability assay and confocal fluorescent microscopy assessed cell growth. Alkaline Phosphatase (ALP) assay and Quantitative Polymerase Chain Reaction (qPCR) examined cell differentiation. Extracellular matrix (ECM) was stained for collagen and calcium. Scanning Electron Microcopy (SEM) was done on sputter coated discs. RESULTS/ANTICIPATED RESULTS: Measured roughness defined by Rz, maximum peak to valley distance of the sample profile ranged from 2.6-65.1 µm. The addition of printed roughness in the sprouts and rough sprouts group significantly diminished ductility resulting in early strain to failure during tensile testing. Cells adhered and proliferated on discs regardless of roughness group. There was no statistical difference in ALP activity, but qPCR showed that rough groups (sprouts and rough sprouts) had diminished Osteocalcin gene expression at week 2 and 4. The ECM in the rough groups was more resistant to repeated washes and was more extensive with SEM. DISCUSSION/SIGNIFICANCE OF IMPACT: Printing roughness diminished mechanical properties without clear benefit to cell growth. Roughness features were on mesoscale, unlike samples in literature on microscale that increase cell activity. Printed topography may aid in implant fixation and not osseous integration as hypothesized. CONFLICT OF INTEREST DESCRIPTION: Dr. Samual Adams, Dr. Ken Gall and Cambre Kelly own stock and/or stock options in restor3d, Inc.

